# Effect of Solvents on the Structure of the Gut Microbiota of Honeybees (*Apis mellifera*)

**DOI:** 10.3390/insects16111076

**Published:** 2025-10-22

**Authors:** Kang Wang, Jinmeng Ma, Ting Ji, Haibo Zhang, Ling Yin

**Affiliations:** 1 College of Animal Science and Technology, Yangzhou University, Yangzhou 225009, China; 2 School of Food Science and Technology, Jiangsu Agri-Animal Husbandry Vocational College, Taizhou 225300, China

**Keywords:** honeybee, cosolvent, gut microbiota, DMSO, DMF, acetone, Tween 80

## Abstract

The gut microbiota of social bees is vital for host health, yet pesticide exposure can disrupt these communities. Because most active ingredients are poorly soluble, toxicological tests often use cosolvents, but their effects remain unclear. We assessed four common cosolvents (DMSO, DMF, acetone, and Tween 80) at laboratory-relevant concentrations on honeybee survival, pollen consumption, body weight, and gut microbiota. In parallel, in vitro assays tested their impact on five dominant gut symbionts. The results showed no significant changes in survival, feeding, body weight, bacterial load, community composition, or core taxa abundance. Similarly, cosolvents did not inhibit bacterial growth in vitro. These findings demonstrate that commonly used cosolvents exert no detectable influence on honeybee physiology or gut microbiota. Although negative, this evidence is critical: it rules out cosolvents as hidden confounders, improving confidence in pesticide toxicology studies and providing essential reference data for pollinator risk assessment.

## Simple Summary

Honeybee gut microbiota is crucial for host health and has been widely studied in the context of pesticide exposure. However, toxicological tests typically require cosolvents to dissolve poorly soluble compounds, raising concerns that these additives might confound results. In this study, we demonstrate that four commonly used cosolvents, at concentrations typical of laboratory assays, have no detectable impact on honeybee survival, physiology, or gut microbiota. Although negative, these results are highly informative: they rule out cosolvents as hidden drivers of experimental outcomes, thereby increasing confidence in the interpretation of pesticide toxicology studies. By clarifying the role of formulation components beyond active ingredients, this work provides essential reference data and strengthens the methodological foundation for pollinator risk assessment.

## 1. Introduction

Honeybees (*Apis mellifera*) play a crucial role as pollinators in both agricultural and natural ecosystems, contributing substantially to biodiversity maintenance and global food production. However, global honeybee populations have been experiencing declines attributed to multiple stressors, including pathogen infections, habitat degradation, and pesticide exposure [1,2]. Among these factors, pesticides are of particular concern due to their extensive use and potential to harm pollinators directly through toxicity or indirectly through sublethal and chronic effects [3].

While the active ingredients in pesticides are often the primary focus of ecotoxicological research, pesticide formulations frequently contain cosolvents and adjuvants to enhance the solubility, stability, and delivery of active compounds [4]. In commercial products, the specific composition of adjuvants and cosolvents often varies between manufacturers and typically involves complex mixtures of chemical compounds [5,6]. Researchers commonly focus on evaluating the biological effects of active ingredients in isolation. Yet, because many active ingredients exhibit poor water solubility, solvents are typically required to ensure proper dissolution. In toxicological experiments, the use of complex formulations is generally avoided, and a single cosolvent is selected to dissolve the active ingredient in order to minimize variability and exclude confounding influences from extraneous formulation components [4,7]. Given the widespread use of cosolvents in pesticides and their potential to affect non-target organisms, it is crucial to evaluate their specific impacts on honeybee health. Ignoring the potential toxicity of cosolvents can result in an inaccurate assessment of the safety profiles of commercial pesticide formulations [8]. Formulation components such as adjuvants and cosolvents, although often considered inert, can markedly alter pesticide toxicity in honeybees [9]. Studies have shown that these compounds may independently cause mortality, interact synergistically with co-occurring stressors, and disrupt colony-level function [9,10]. In extreme cases, the overall toxicity of formulated products, particularly fungicides, has been reported to exceed that of the active ingredients by several orders of magnitude [11]. Observed effects include impaired cognitive performance in adult bees, chronic developmental toxicity in larvae, reduced sucrose consumption, and weight loss [12,13], indicating that non-active formulation constituents warrant greater consideration in pollinator risk assessments.

In laboratory toxicology testing of honeybees, pesticide active ingredients are often dissolved in cosolvents due to their limited water solubility. According to the COLOSS BEEBOOK [14] and OECD Test Guideline No. 245 [15], acetone is the preferred solvent for topical and oral tests, while DMSO and DMF are accepted low-toxicity alternatives when compounds are not soluble in acetone. For highly polar or water-dispersible substances, non-ionic surfactants (such as Tween 80), which can also act as cosolvents, are recommended as suitable adjuvants. Despite their widespread use, the potential effects of these cosolvents on non-target organisms, such as honeybees, particularly on the gut microbiota, have received limited attention. The gut microbiota of honeybees plays a vital role in maintaining host health, contributing to nutrient metabolism, immune modulation, and protection against pathogens [16,17,18]. However, recent years have seen an increasing number of studies reporting disruptions to honeybee gut microbial communities caused by various pesticides [19], many of which were formulated using cosolvents, highlighting the need to consider not only the active ingredients but also the potential impacts of cosolvent components. This study aimed to evaluate the effects of four commonly used cosolvents—namely dimethyl sulfoxide (DMSO), N,N-dimethylformamide (DMF), acetone, and Tween 80, which are frequently applied as solvents in honeybee toxicological studies [20,21]—on honeybee survival, pollen consumption, body weight, and gut microbiota composition. The objective was to disentangle cosolvent effects from those of active ingredients in order to provide direct evidence of their influence on honeybee physiology and gut microbial communities. By clarifying whether these solvents exert detectable impacts at laboratory-relevant concentrations, this work offers essential reference data for toxicological study design and contributes to more reliable risk assessments of pesticide formulations and pollinator health.

## 2. Materials and Methods

### 2.1. Honeybee and Chemicals

The experimental design followed the OECD Test Guideline No. 245 for chronic oral toxicity testing of adult honeybees (*Apis mellifera*), which specifies a 10-day exposure period [15], and the experiment was carried out under laboratory conditions. We evaluated the potential effects of four commonly used pesticide cosolvents: DMSO, DMF, acetone, and Tween 80, on the composition and diversity of the gut microbiome in honeybees (*Apis mellifera*). These cosolvents were selected because they are frequently employed in laboratory toxicological studies as solubilizers for pesticide active ingredients [14]. As cosolvents may confound experimental outcomes, their concentrations are typically restricted to low levels in toxicological assays. Accordingly, we selected 0.1%, a concentration commonly used in honeybee toxicological studies [22,23,24], as the test concentration. All pesticide cosolvents were obtained from the laboratory of Jianguo Chen, College of Horticulture and Plant Protection, Yangzhou University.

### 2.2. Effects of Pesticide Cosolvents on Honeybee Survival, Pollen Consumption, and Body Weight

Late-stage pupae with dark eyes were collected and placed in 24-well plates under sterile conditions to emerge in the laboratory. These bees are typically considered germ-free at this stage. Newly emerged worker bees (within 24 h) were then grouped into five experimental groups (*n* = 30 bees per group) and housed in 5 separate cup cages. The bees were maintained in an artificial climate chamber under controlled environmental conditions at 33 °C and 70% relative humidity. All worker bees were provided with sterilized pollen bread (made by mixing dry pollen (m) with 50% sucrose syrup (v) in a 2:1 ratio) supplemented with natural nurse bee gut homogenate for 10 days, a sufficient duration for the establishment of normal gut microbiota, as previously reported [7]. Whole guts were dissected from 5 nurse bees and pooled into a single tube containing 5 mL of sterile 1× phosphate-buffered saline (PBS) and 5 mL of sterile sucrose syrup. The mixture was thoroughly homogenized, and 500 μL of the gut homogenate was added to 1 g of bee bread for microbial inoculation. After day 2, all groups were maintained on sterile bee bread and sucrose syrup without further supplementation of gut homogenate. Control bees were fed 50% sterilized sucrose syrup, and treatment groups were fed the same sucrose syrup containing 0.1% DMSO, DMF, acetone, or Tween 80. Pollen consumption was determined by subtracting the remaining pollen from the total amount provided, with the result then divided by the number of surviving bees in each group to account for mortality. Pollen consumption and mortality were recorded every two days, with deceased individuals removed during food changes. The experiment was repeated five times, resulting in five biological replicates.

On day 10, ten bees were randomly selected; the bee’s entire gut was dissected under sterile conditions for subsequent microbiota analysis. The body weight of each individual bee was measured after the gut was removed.

### 2.3. Quantification and Structural Analysis of the Gut Microbial Community

Total DNA was extracted from the gut using the TIANamp Stool DNA Kit (Tiangen Biotech Co., Ltd., Beijing, China). The bacterial load was quantified by amplifying the 16S rRNA gene with universal bacterial primers [25]. Absolute copy numbers were determined using standard curves derived from the amplification of a cloned target sequence in a pGEM-T vector (Promega, Madison, WI, USA). The final bacterial load for each sample was calculated based on the standard curve and adjusted for dilution.

The V4 region of the 16S rRNA gene was amplified using universal primer pairs, combined with adapter and barcode sequences. PCR products were quantified and purified using the GeneJET Kit, before being pooled for sequencing on the Illumina HiSeq 2500 platform (Novogene Co., Ltd., Beijing, China). Low-quality reads (<0.1% abundance), as well as chloroplast and mitochondrial sequences, were removed using Cutadapt (V1.9.1, http://cutadapt.readthedocs.io/en/stable, 14 September 2025). Clean reads were obtained by removing barcode and primer sequences, and paired reads were merged using Vsearch (V2.15, https://github.com/torognes/vsearch, 14 September 2025). Taxonomic assignment was performed using a local database of honeybee gut bacteria 16S rDNA sequences. Statistical and visualization analyses were conducted using specialized R packages (https://github.com/microbiota, 14 September 2025).

### 2.4. In Vitro Growth Assay of Gut Bacteria Under Cosolvent Exposure

To evaluate whether pesticide cosolvents have direct antimicrobial effects on honeybee gut symbionts, five dominant bacterial strains (*Lactobacillus* Firm-5, *Bombilactobacillus* Firm-4, *Bifidobacterium*, *Gilliamella*, and *Snodgrassella alvi*) were subjected to in vitro growth assays under cosolvent exposure. All strains were obtained from the Culture Collection Centre of the Institute of Apiculture Research at Yangzhou University. *L*. Firm-5, *B*. Firm-4, and Bifidobacterium were cultured on MRS agar under anaerobic conditions, whereas *Gilliamella* and *S. alvi* were cultured on TSA agar in an atmosphere containing 5% CO_2_; incubation was performed at 35 °C until isolated colonies appeared. Single colonies were subsequently transferred into the corresponding liquid medium (MRS broth for *Lactobacillus* and *Bifidobacterium*; TSA broth for *Gilliamella* and *Snodgrassella*) and grown overnight. The optical density of each culture was then adjusted to OD_600_ = 1.0 with fresh medium to standardize the inoculum concentration across strains. Standardized suspensions were inoculated into sterile 96-well microplates, with each well containing 200 μL of broth supplemented with one of three cosolvents (DMSO, DMF, or acetone) at a final concentration of 0.1% (*v*/*v*). Tween 80 was not included, as it forms a turbid emulsion that interferes with optical density measurements. A positive control group was prepared by adding tetracycline (20 μg/mL) to the medium to assess growth inhibition. Cultures were incubated at 35 °C for 48 h under the respective atmospheric conditions, and OD_600_ (optical density at 600 nm) was recorded every 12 h using a Tecan^®^ microplate reader after 2 min of gentle shaking. All treatments were conducted in triplicate, and data are presented as mean OD_600_ values ± standard deviation.

### 2.5. Statistical Analysis

Kaplan–Meier survival curves were generated over a 10-day period, and group differences were assessed using the log-rank test. Differences in pollen consumption, body weight, gut microbial abundance, and alpha diversity indices (Shannon index) between groups were analyzed using the Mann–Whitney *U* test. Differences in community composition among treatment groups were evaluated using PERMANOVA with the adonis function in the vegan package (version 2.6-4) in R software (version 4.3.2). Statistical significance is indicated as follows: *, *p* < 0.05; **, *p* < 0.01; and ***, *p* < 0.001.

## 3. Results

### 3.1. Cosolvent Exposure Does Not Significantly Affect Honeybee Survival, Pollen Consumption, or Body Weight

**Figure 1:**
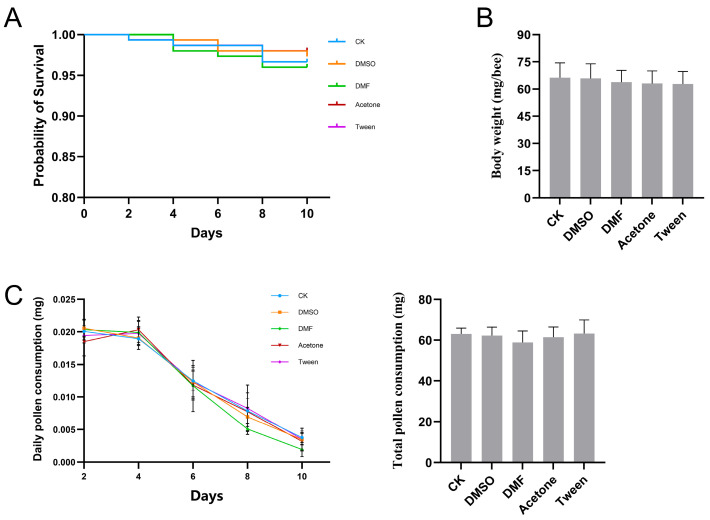
Effects of exposure to cosolvents on honeybee survival, body weight, and pollen consumption. (**A**) Kaplan–Meier survival curves over 10 days, showing no significant differences among control (CK) and treatment groups; *n* = 150. (**B**) Body weight measured post gut removal, showing no significant differences among groups; *n* = 50. (**C**) Daily and total pollen consumption per bee showed no significant differences among treatment groups; *n* = 150.

Our results showed that exposure to pesticide cosolvents (DMSO, DMF, acetone, and Tween 80) at a concentration of 0.1% did not result in significant honeybee mortality (*p* = 0.877; Figure 1A). Pollen consumption during the 10-day exposure period and final body weight were also assessed. No significant differences were observed in either final body weight (Figure 1B; *p* = 0.423) or total pollen consumption (Figure 1C; *p* = 0.598) between the cosolvent exposed and control groups.

### 3.2. In Vivo Exposure to Cosolvents Does Not Significantly Alter Gut Microbiome Size or Composition

**Figure 2:**
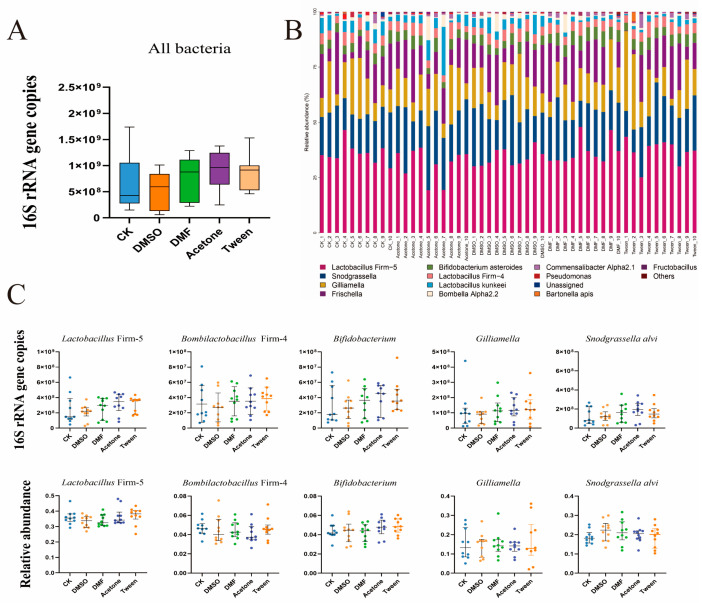
Effects of pesticide cosolvent exposure on honeybee gut microbiome size and composition. (**A**) Total bacterial load quantified as 16S rRNA gene copy number, showing no significant differences among control (CK) and treatment groups. (**B**) Relative abundance of gut bacterial taxa, indicating no major compositional shifts among groups. (**C**) Absolute copy number of core bacterial taxa showing no significant differences among groups. n = 10.

Newly emerged worker bees were simultaneously exposed to DMSO, DMF, acetone, and Tween 80. No evidence was found indicating that the four pesticide cosolvents disrupted the honeybee gut microbiome. There were no significant changes in gut bacterial load (Figure 2A, *p* = 0.423), community composition (Figure 2B), or the absolute and relative abundance of five core taxa (Figure 2C, *p* > 0.05).

**Figure 3:**
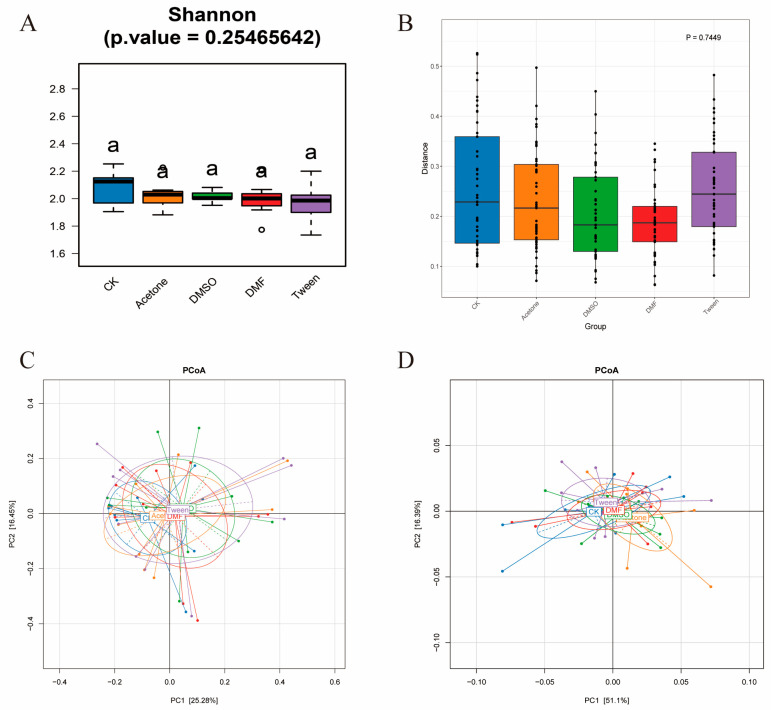
Effects of pesticide cosolvent exposure on honeybee gut microbiome diversity. (**A**) Shannon index showing no significant differences among groups, Bars sharing the same letter (a) are not significantly different (*p* > 0.05). (**B**) Beta diversity based on Bray–Curtis dissimilarities, with no significant variation detected among groups as assessed by PERMANOVA. (**C**,**D**) PCoA plot (weighted and unweighted Bray–Curtis) showing high overlap in community structures.

We further analyzed the gut microbial community structure in bee guts. Based on relative abundance metrics, pesticide cosolvent exposure did not result in significant changes in microbial diversity within individual hosts (alpha diversity, assessed using the Shannon index, Figure 3A, *p* = 0.254). Similarly, no significant differences in overall community composition were observed between treatment groups (beta diversity, assessed using Bray–Curtis dissimilarity, Figure 3B; PERMANOVA, *p* = 0.744. Principal coordinate analysis (PCoA) based on weighted and unweighted Bray–Curtis distances did not reveal distinct clustering among treatment groups (Figure 3C,D). This observation is consistent with the PERMANOVA results (*p* = 0.7449; Figure 3B), indicating that exposure to 0.1% cosolvents had no significant impact on gut microbial community structure.

### 3.3. In Vitro Exposure to Pesticide Cosolvents Does Not Inhibit the Growth of Dominant Gut Symbionts

**Figure 4:**
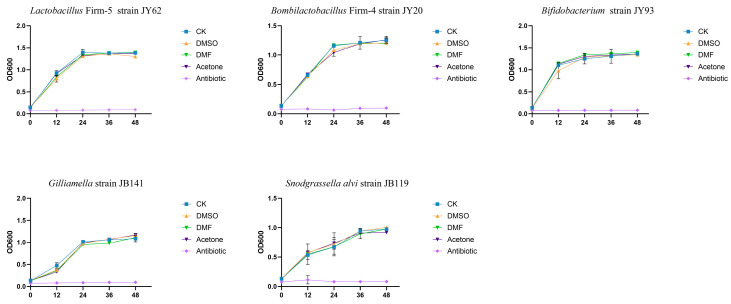
In vitro growth of dominant honeybee gut symbionts under pesticide cosolvent exposure. Growth curves of *Lactobacillus* Firm-5 (strain JY62), *Bombilactobacillus* Firm-4 (strain JY20), *Bifidobacterium* (strain JY93), *Gilliamella* (strain JB141), and *Snodgrassella alvi* (strain JB119) in broth supplemented with 0.1% dimethyl sulfoxide (DMSO); N, N-dimethylformamide (DMF); or acetone. Sterile water was used as a negative control, and tetracycline (20 μg/mL) served as an antibiotic control. Data are shown as mean ± standard deviation.

In vitro growth assays were performed to determine whether the tested cosolvents exert direct antimicrobial effects on dominant honeybee gut symbionts. All five strains, including *Lactobacillus* Firm-5, *Bombilactobacillus* Firm-4, *Bifidobacterium*, *Gilliamella*, and *Snodgrassella alvi*, showed comparable growth dynamics in the control medium and in medium supplemented with 0.1% DMSO, DMF, or acetone (Figure 4). The OD_600_ values increased steadily during the incubation period, and no significant differences were observed between cosolvent-treated and untreated groups. By contrast, the addition of tetracycline (20 µg/mL) led to a marked suppression of bacterial growth in all strains, confirming the sensitivity of the assay. These results demonstrate that DMSO, DMF, and acetone at 0.1% do not inhibit the proliferation of the major honeybee gut symbionts under the conditions tested.

## 4. Discussion

This study presents the first systematic assessment of four carrier cosolvents commonly used in honeybee toxicological research (DMSO, DMF, acetone, and Tween 80) tested at 0.1%, a concentration widely applied in laboratory assays. The results clearly showed that these solvents exerted no measurable effects on adult worker survival, feeding, or body weight, nor did they alter the gut bacterial load or community composition relative to controls.

In recent years, researchers have increasingly recognized the importance of so-called “inert” ingredients in honeybee toxicology [11]. However, this issue remains challenging because commercial pesticide formulations, though more representative of field exposure, differ considerably among manufacturers in both the proportion and chemical composition of non-active constituents. Moreover, detailed information on these formulation components is rarely disclosed, as they are often considered proprietary trade secrets, which complicates efforts to evaluate their specific impacts on bee health. Several studies have reported that formulated products can be more toxic than their active ingredients alone and can exert pronounced effects on honeybee physiology and behavior [10,11,13,26,27]. Under controlled laboratory conditions, however, researchers typically focus on the active ingredients themselves and use cosolvents mainly to enhance solubility and achieve target concentrations. This highlights the need to evaluate the potential toxicological effects of these solvents, rather than assuming they are biologically inert. In practice, cosolvents are selected for their relatively low intrinsic toxicity, which makes them suitable carriers for experimental use. For example, acetone is widely accepted in honeybee toxicological studies and is permitted under international regulatory guidelines. At low concentrations, it produces minimal adverse effects, whereas its toxicity increases sharply with dose. Chronic exposure studies indicate that 0.1–0.5% acetone has negligible effects on adult bee mortality and feeding, while concentrations of 2% or higher significantly increase mortality and reduce food intake. Similarly, 22-day larval assays identify 0.5% acetone as safe, but higher levels markedly reduce survival [28]. Recent research combining in vitro and in vivo larval rearing with RFID tracking further showed that 0.1% acetone did not influence larval development, although adult exposure shortened lifespan and altered foraging behavior in a season-dependent manner [29]. When applied at 1.5%, acetone produced outcomes comparable to water-treated controls, supporting its general suitability as a low-toxicity carrier. Nevertheless, concentrations of 5–10% in larval diets significantly increased mortality [30], and even low topical doses reduced sucrose responsiveness in newly emerged and foraging honeybees [31]. Together, these findings demonstrate that cosolvents toxicity is highly dose-dependent and context-specific.

Other organic cosolvents such as DMSO and DMF are also widely used as carriers for poorly soluble compounds in biological assays. However, mounting evidence indicates that they may influence bacterial physiology at elevated concentrations. For instance, DMSO above 5% can alter bacterial growth and gene expression [32] and interfere with antibiotic-induced killing in *Escherichia coli* [33]. Similarly, DMF can be metabolized or degraded by certain bacteria such as *Paracoccus* species and biofilm-forming strains, indicating species-specific tolerance differences [34,35]. Acetone, DMF, and DMSO have also been reported to disrupt microbial membranes and impair nutrient uptake through changes in membrane fluidity [36,37,38]. Nonetheless, some microorganisms, particularly Gram-negative species, exhibit remarkable tolerance to organic solvents because their outer membrane restricts the entry of hydrophobic compounds [39,40]. For example, *Pseudomonas putida* strains can actively grow in media containing up to 50% (*v*/*v*) toluene [36,41,42].

Our results demonstrate that commonly used cosolvents did not affect bacterial growth in vitro and caused no dysbiosis of the honeybee gut microbiota in vivo. Importantly, both assays yielded consistent outcomes, indicating that these solvents at the tested concentrations have negligible impact on gut symbionts. In contrast, Praet et al. (2021) reported discrepancies between in vitro and in vivo assays, where oxalic acid suppressed certain strains under laboratory conditions but induced more pronounced shifts in community composition in vivo [43]. These differences highlight the importance of assessing solvent effects in both experimental contexts. By combining in vitro growth assays with in vivo exposure trials, our study provides robust evidence that cosolvents do not interfere with honeybee gut bacteria.

## 5. Conclusions

The gut microbiota of social bees is essential for host health and performance, and growing evidence shows that pesticide exposure can disrupt these microbial communities [19,43,44]. Nevertheless, the contribution of cosolvents to observed outcomes has remained largely unexamined. Our results demonstrate that four commonly used cosolvents (DMSO, DMF, acetone, and Tween 80), at typical laboratory concentrations of 0.1%, are not major drivers of gut dysbiosis, highlighting the need to focus pesticide research on active ingredients. These findings provide a valuable reference for toxicological assays and underscore the importance of risk assessments that consider the combined effects of all formulation components; we also stress that researchers should report formulation details and evaluate novel solvents or adjuvants on a case-by-case basis. It is important to note, however, that the cosolvent selection and exposure methods employed here reflect standardized laboratory toxicological testing rather than field-realistic scenarios and that no positive control was included in the in vivo assay, which we acknowledge as a methodological limitation. Future studies should therefore examine multiple concentrations and chronic, environmentally relevant exposures and include appropriate positive controls to fully assess the potential role of cosolvents in pollinator health.

## Data Availability

The raw sequence data reported in this paper have been deposited in the Genome Sequence Archive in the National Genomics Data Center, China National Center for Bioinformation/Beijing Institute of Genomics, Chinese Academy of Sciences (GSA: CRA026244). The data are publicly accessible at https://ngdc.cncb.ac.cn/gsa (14 September 2025).

## Abbreviations

The following abbreviations are used in this manuscript:

DMSO	Dimethyl sulfoxide
DMF	Dimethylformamide
OD_600_	Optical density at 600 nm
PCoA	Principal coordinate analysis
PERMANOVA	Permutational multivariate analysis of variance
